# Laryngoscope based lighted stylet for intubation: An innovation

**DOI:** 10.4103/1658-354X.71575

**Published:** 2010

**Authors:** Harsimran Singh, Anurag Tewari, Abishek Bansal, Balvinder Kaur

**Affiliations:** *Department of Anaesthesia, Dayanand Medical College and Hospital, Ludhiana, Punjab, India*

Sir,

Tracheal intubation is older than the recognized history of general anesthesia itself. In the late 18th century, the royal humane society of London used tracheal intubation for resuscitating the near-drowned.[1] Laryngoscopy and endotracheal intubation are both intensely stimulating procedures and are associated with varying degrees of sympathetic activity which may be detrimental in patients with coexisting conditions, such as coronary artery disease, elevated intracranial pressure, and asthma. Several groups have investigated the possibility that lighted stylet intubation may result in less stimulation than direct laryngoscopy and may offer some protective effects from sympathetic hyperactivity.[2]

The first reported use of a lighted stylet used to facilitate intubation came in 1957 when Sir Robert Macintosh[3] described an 18-inch illuminated tracheal tube introducer, which was designed to stiffen the tube. Over the past few decades, light-guided intubation using the principle of trans-illumination has proven to be an effective, safe, and simple technique. A lighted stylet uses the principle of trans-illumination of the soft tissues of the anterior neck to guide the tip of the endotracheal tube into the trachea. This technique takes advantage of the anterior or more superficial location of the trachea in relation to the esophagus.[4]

**Figure 1:**
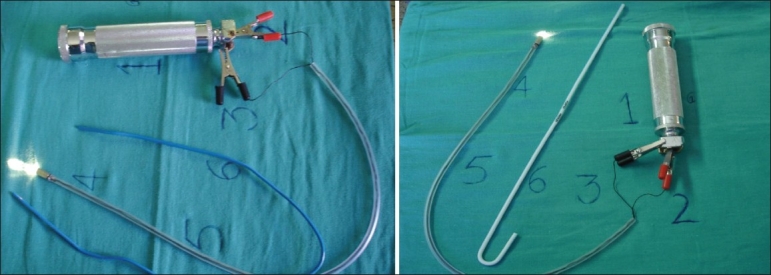
Laryngoscope-based lighted stylet showing the wires are connected with the connecting hooks to the handle of the laryngoscope.

Current lightwands are largely fiberoptic in design and have either external or internal light sources. Newer devices have been developed that use trans-illumination or transmission of sound, in combination with other intubating guides. The newer ones combine a lighted stylet with a fiberoptic scope. This combination is intended to provide trans-illumination and internal visualization of the larynx either directly or on a screen.[5] The cost of such expensive newer instruments is the main reason for their non-usage in poor third world countries. We have designed an indigenous laryngoscope-based lighted stylet. It comprises the outer tubing of the suction catheter through which the normal 2mm thin stylet can be passed. It has two thin wires also passing through it. At the distal end the bulb used in laryngoscope is soldered with the stylet and the wires and fixed with a permanent binding material. The endotracheal tube of appropriate size is loaded onto it. The wires are connected with the connecting hooks to the handle of the laryngoscope. Our innovatively designed laryngoscope-based lighted stylet uses only the freely available, inexpensive material and perhaps is the only stylet which does not use any additional light source [Figure 1].

Lighted stylet tracheal intubation requires practice, but is easy to learn. The distal end of the stylet should be lubricated water-based surgical lubricant and the tracheal tube loaded onto it. For oral intubation, the nondominant hand is used to open the mouth with the thumb placed against the mandibular molars while the opposing index finger is pressed against the ramus of the mandible; this hand should be kept as far lateral as possible to allow unobstructed midline placement of the lighted stylet. A firm anterocaudad jaw thrust elevates the epiglottis, improves tactile sensation, and facilitates intubation via gentle lighted stylet motions with the dominant hand. The lighted stylet is introduced into the oropharynx from the side and brought into the midline following the midsagittal plane transecting the tongue. The tip of the lightwand should be passed under the tongue, and with gentle anterior traction, a bright well circumscribed circle of light at the level of the hyoid indicates that the tip lies in the vallecula. Gentle forward pressure may displace the epiglottis, allowing immediate tracheal intubation. If a bright red glow is seen off the midline, then the tip of the lightwand may lie in one of the pyriform fossae, and the unit should be withdrawn slightly and rotated back toward the midline. If resistance is felt preventing passage into the trachea, then the obstructing epiglottis may be circumvented by a series of rocking or scooping movements redirecting the tip to the thyroid prominence by using the light as a guide after careful withdrawal of the stylet following the contour of the oropharynx, the 15-mm adapter is reconnected and the tracheal tube placement is confirmed in the usual manner.[1]

There are chances of the dislodgement of the bulb attached at the distal end while removing the lighted stylet

The lighted stylet can be used only for adult patients.

The bulb might get heated and may be a source of thermal injury and the possibility of heat damage to the tracheal mucosa during prolonged intubation attempts should be kept in mind.

Minor trauma to upper airway leading to bleeding, sore throat, hoarseness, and dysphagia

The most severe upper airway damages reported after lighted stylet intubation are reported cases of arytenoid cartilage dislocation.
